# Risk factors affecting perioperative analgesic efficacy in transurethral resection of the prostate and the impact of mind map—guided nursing on pain management: a retrospective study

**DOI:** 10.3389/fsurg.2025.1555251

**Published:** 2025-09-25

**Authors:** Huifeng Wu, Biyun Wen, Jiaqian Liu, Yanfang Yu

**Affiliations:** The Eighth Affiliated Hospital, Southern Medical University (The First People's Hospital of Shunde, Foshan), Foshan, Guangdong, China

**Keywords:** transurethral resection of the prostate (TURP), analgesic efficacy, mind map-guided nursing, pain management, nursing intervention

## Abstract

**Purpose:**

This study aimed to analyze the factors influencing perioperative analgesic efficacy in Transurethral Resection of the Prostate (TURP) and evaluate the effectiveness of mind map-guided nursing interventions in reducing perioperative pain and improving nursing quality.

**Methods:**

A retrospective analysis was conducted on clinical data from 140 patients who underwent TURP surgery at our institution between January and December 2023. Following PRISMA guidelines, patients were systematically screened and stratified into two groups based on analgesic efficacy 72h post-surgery: good analgesic response group (Visual Analogue Scale [VAS] score ≤6) and poor analgesic response group (VAS score >6). Univariate analysis was performed on clinical parameters, followed by multivariate logistic regression analysis on statistically significant factors to identify independent risk factors affecting perioperative analgesic efficacy. To address potential multicollinearity between ASA classification and comorbidities, variance inflation factor (VIF) analysis was conducted. Subsequently, 80 patients with poor analgesic response were randomized into two equal groups (*n* = 40 each): an observation group receiving mind map-guided nursing intervention and a control group receiving standard nursing care. Outcome measures included VAS scores, psychological status (anxiety and depression), sleep quality, urinary incontinence severity, and nursing satisfaction. The certainty of evidence was assessed using the GRADE approach.

**Results:**

Of the 152 eligible patients screened, 140 met inclusion criteria and were analyzed. Univariate analysis revealed that the poor analgesic response group had significantly longer operation times and higher proportions of American Society of Anesthesiologists (ASA) class II patients, smokers, and individuals with hypertension or diabetes (all *P* < 0.05). Multivariate logistic regression identified prolonged operation time (OR = 1.528, 95% CI: 1.218–1.982) and smoking history (OR = 1.278, 95% CI: 1.042–1.826) as independent risk factors for poor perioperative analgesic response. ROC curve analysis demonstrated good predictive accuracy (AUC = 0.782, 95% CI: 0.705–0.859). The mind map-guided intervention group demonstrated significantly lower post-intervention scores for pain (VAS), anxiety, depression, sleep disturbance, and urinary incontinence compared to the control group (all *P* < 0.05). Additionally, nursing satisfaction rates were significantly higher in the intervention group (95.00% vs. 75.00%, *P* < 0.05).

**Conclusion:**

Perioperative analgesic efficacy in TURP patients is significantly influenced by operation duration and smoking history as independent risk factors. Mind map-guided nursing intervention effectively reduces postoperative pain, improves psychological outcomes, and enhances patient satisfaction, warranting its broader clinical implementation.

## Introduction

Benign Prostatic Hyperplasia (BPH) represents one of the most prevalent urological conditions affecting elderly men globally. The incidence of BPH demonstrates a strong age-dependent correlation, with prevalence rates exceeding 50% in men aged 60 years and reaching approximately 83% by age 80 (1–3). Transurethral Resection of the Prostate (TURP) has established itself as the gold standard surgical intervention for BPH management, offering advantages such as minimal invasiveness, rapid recovery, and broad applicability (4, 5). However, postoperative pain management remains a significant clinical challenge despite the advancement of analgesic techniques and pharmacological interventions, including preemptive analgesia and local ropivacaine administration (6, 7).

The evolution of enhanced recovery after surgery (ERAS) protocols has heightened awareness regarding the importance of effective postoperative pain management in TURP patients. Identifying patients at high risk for inadequate pain control has become crucial for implementing personalized treatment strategies (8, 9). Although there have been various efforts in pain management for TURP patients, existing strategies often fall short. This is because they typically do not comprehensively account for the complex interaction of patient—specific factors. For instance, diabetes can compromise immune system function and metabolic regulation. In cases of long—term illness or poor glycemic control, it may lead to a reduced response to standard analgesic protocols. Hypertensive patients often experience vascular endothelial dysfunction, which affects their ability to tolerate surgical stress and respond to pain management. Smoking, due to nicotine's action on specific receptors like nicotinic acetylcholine receptors, can cause desensitization of analgesic responses, increasing the analgesic requirements. As a result, the effectiveness of current pain management strategies is limited, and there is a need for more targeted approaches. Despite this recognized importance, research examining factors associated with postoperative analgesic efficacy remains limited. To address this knowledge gap, our study conducted a retrospective analysis of clinical data from 140 TURP patients to investigate factors influencing postoperative analgesic outcomes, aiming to develop targeted and effective pain management strategies.

Current literature reveals numerous studies examining perioperative nursing interventions for BPH patients, particularly focusing on pain management and psychological support. However, the effectiveness of these interventions has often fallen short of clinical expectations (10, 11). Mind mapping, originally developed by Tony Buzan in the 1970s, has been successfully implemented in various healthcare settings including nursing education, clinical decision-making, and patient care planning (12, 13). This visual thinking tool has demonstrated effectiveness in improving information retention, enhancing critical thinking, and facilitating communication between healthcare providers and patients (14). In the context of nursing care, mind map-guided interventions have shown promise in managing chronic conditions, postoperative recovery, and patient education across different surgical specialties (15, 16). Recent evidence suggests that mind map-guided nursing interventions can enhance nursing quality, ameliorate pain symptoms, and improve patients' quality of life (12). Building on these findings, our study selected 80 patients with suboptimal perioperative analgesic responses to TURP to evaluate the efficacy of mind map-guided nursing interventions, seeking to establish evidence-based recommendations for pain management optimization.

Our study aim to identify the independent risk factors influencing perioperative analgesic efficacy in TURP patients through univariate and multivariate analyses. The secondary objectives are to evaluate the impact of mind map—guided nursing interventions on pain intensity [assessed by Visual Analogue Scale (VAS) scores], psychological status [measured by Self—rating Anxiety Scale (SAS) and Self—rating Depression Scale (SDS)], sleep quality, urinary incontinence severity, and nursing satisfaction.

## Materials and methods

### Study design and patient population

This is a retrospective, randomized study. The study was conducted following the Strengthening the Reporting of Observational Studies in Epidemiology (STROBE) guidelines for retrospective cohort studies. We analyzed clinical data from 140 patients who underwent TURP at the Shunde Hospital of Southern Medical University between January and December 2023. This retrospective data collection allowed us to gather information on various patient characteristics and surgical outcomes. Based on the analgesic efficacy 72 h post—surgery, patients were stratified into two groups: good analgesic response group [Visual Analogue Scale (VAS) score ≤ 6; *n* = 60] and poor analgesic response group (VAS score > 6; *n* = 80).

Before randomization, all patients received the standard preoperative education and routine perioperative management as part of the general hospital care protocol. This was to ensure a consistent baseline for all patients before the different nursing interventions were implemented.

After identifying the 80 patients in the poor analgesic response group, we employed a computer—generated sequence for randomization. This randomization process was used to assign these patients into two equal groups (*n* = 40 each). One group was designated as the observation group, which received mind—map guided nursing intervention, and the other as the control group, which received standard nursing care. This random assignment was crucial for evaluating the efficacy of the nursing interventions.

### Patient screening process

**Figure 1:**
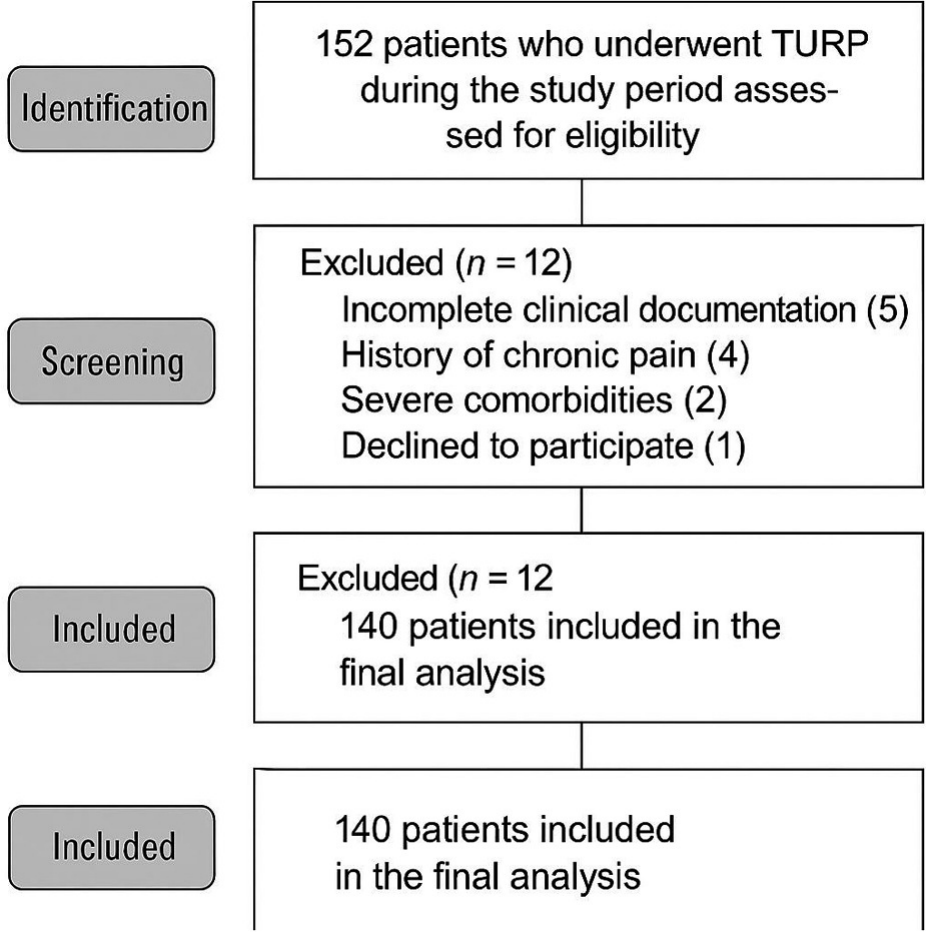
CONSORT Flow Diagram of Patient Selection and Randomization.

Figure 1 illustrates the patient screening flowchart. Initially, 152 patients who underwent TURP during the study period were assessed for eligibility. Of these, 12 patients were excluded: 5 had incomplete clinical documentation, 4 had a history of chronic pain, 2 had severe comorbidities, and 1 declined to participate. The remaining 140 patients were included in the final analysis.

### Inclusion and exclusion criteria

Inclusion criteria encompassed: Prostatic hyperplasia patients with TURP indication, no surgical contraindications, undergoing primary TURP, having complete clinical documentation, and providing written informed consent.

Exclusion criteria comprised: History of chronic pain, severe cardiac/hepatic/pulmonary/renal comorbidities, narcotic dependence or substance abuse, disease recurrence/chronic infection/previous TURP, and unwillingness to participate. The study protocol was approved by the Medical Ethics Committee of the Eighth Affiliated Hospital, Southern Medical University (The First People's Hospital of Shunde, Foshan) (Certificate number: KYLS20220714).

### Search strategy and literature review

For the background and rationale of mind map-guided nursing interventions, we conducted a comprehensive literature search. The search was performed in PubMed, EMBASE, and CNKI databases from inception to December 2023. Search terms included: (“mind map” OR “mind mapping” OR “concept map”) AND (“nursing” OR “nurse-led” OR “nursing intervention”) AND (“pain management” OR “postoperative pain” OR “analgesic”). No language restrictions were applied. The last search was conducted on December 15, 2023.

### Intervention methods

The control group received standard nursing care, including preoperative education and routine perioperative management (13).

The observation group received mind map-guided nursing intervention, which consisted of two primary components:

### Mind map development

(a) Healthcare team members first collected comprehensive patient information, including medical history, surgical details, pain tolerance levels, and psychological status. Based on this information, they identified key nursing elements relevant to each patient. For example, for a patient with a history of diabetes, the importance of blood sugar control during postoperative recovery was emphasized; for a patient with high anxiety, specific relaxation techniques were included. These elements were then incorporated into patient—specific nursing protocols.
(b) Created hierarchical visual representations combining key nursing elements with relevant images
(c) Conducted interdisciplinary reviews and refinements through team discussions
(d) Incorporated evidence-based practices through literature review
(e) Distributed compiled mind maps to patients for reference

### Mind map implementation

(a) Comprehensive pain assessment protocols
(b) Standardized evaluation tools (VAS, numerical rating scales)
(c) Systematic analysis of pain etiology
(d) Individualized intervention strategies
(e) Family education regarding supportive care
(f) Psychological support mechanisms (14, 15)
(g) All nurses involved in the study received specialized training. The training covered the principles of mind map construction, how to use mind maps for patient education and assessment, and how to implement individualized intervention strategies based on the mind map content. They were also trained on communication skills to effectively explain the mind map to patients and answer their questions.

**Figure 2:**
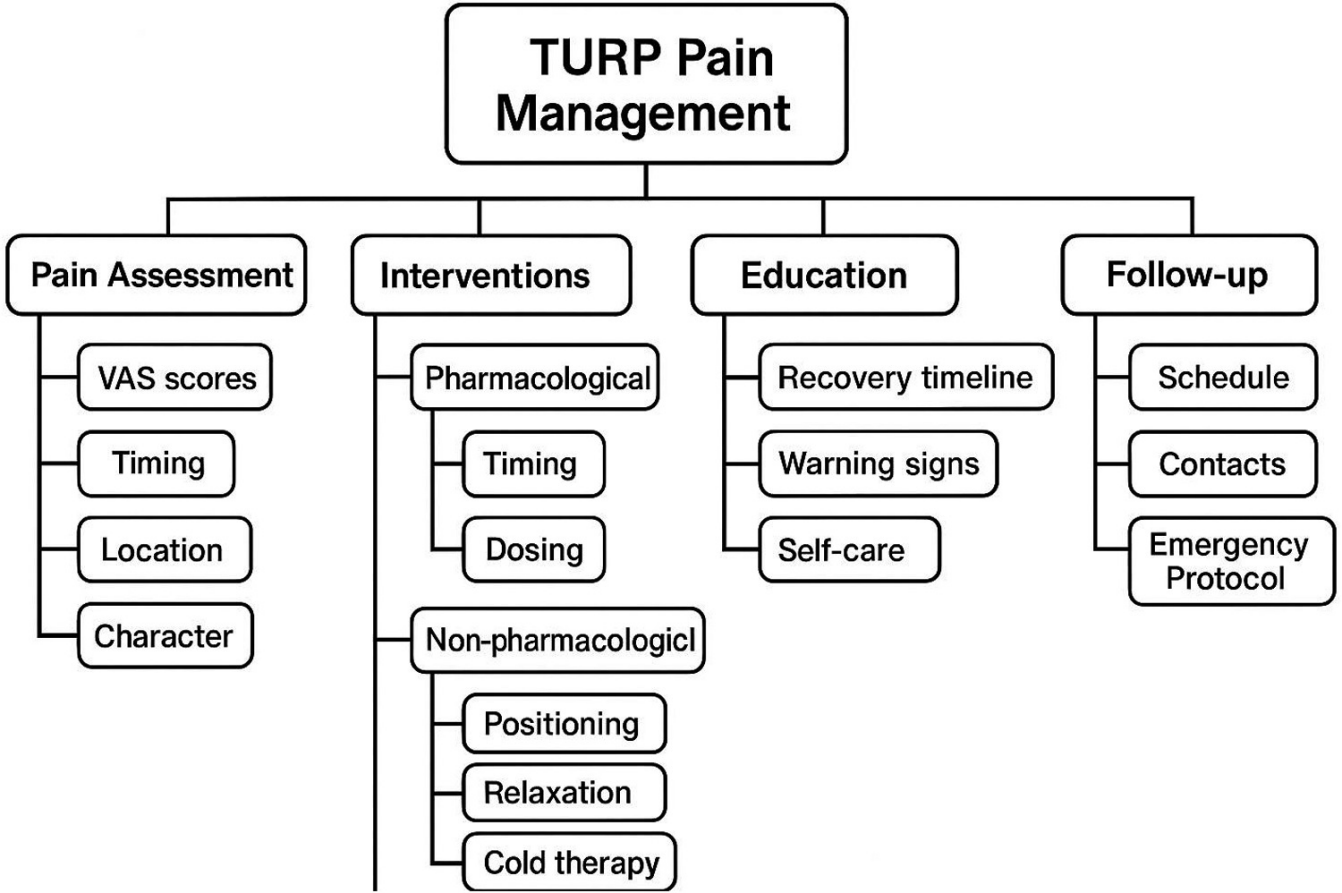
Example of Mind Map for TURP Pain Management.

Figure 2 Example of Mind Map-Guided Nursing Intervention for TURP Patients [A detailed mind map would be included here showing the central concept “TURP Pain Management” with branches covering: (1) pain Assessment (VAS scores, timing, characteristics), (2) pharmacological interventions (analgesics, timing, dosing), (3) non-pharmacological interventions (positioning, relaxation techniques, cold therapy), (4) patient education (expected recovery, warning signs, self-care), (5) psychological support (anxiety management, family involvement, coping strategies), and (6) follow-up care (monitoring schedule, contact information, emergency protocols)].

The fundamental differences between mind map-guided nursing and conventional nursing include: (1) visual representation of care protocols enhancing patient understanding and retention; (2) individualized content based on patient-specific risk factors and needs; (3) structured yet flexible framework allowing for dynamic adjustments; (4) enhanced patient engagement through interactive visual tools; (5) standardized training ensuring consistent implementation across nursing staff.

### Outcome measures

1. Univariate Analysis: Clinical parameters included age, gender, body mass index (BMI), operation duration, American Society of Anesthesiologists (ASA) classification, analgesia method, and comorbidities. To address potential multicollinearity between ASA classification and comorbidities (hypertension and diabetes), we calculated variance inflation factors (VIF) for all variables. Variables with VIF >5 were considered to have multicollinearity issues.
  Multivariate Analysis: Statistically significant variables from univariate analysis underwent logistic regression to identify independent risk factors.
  Predictive Model Development: Based on the identified risk factors, we developed a predictive model and evaluated its performance using receiver operating characteristic (ROC) curve analysis. The area under the curve (AUC), sensitivity, specificity, and optimal cut-off values were calculated.
  Pain Assessment: VAS scores were assessed at two time points: pre—intervention (before the start of any nursing interventions) and 72 h post—intervention (16).

### Psychological evaluation

Anxiety: Self-rating Anxiety Scale (SAS) comprising 20 items (score range: 20–80; ≥50 indicating anxiety)
Depression: Self-rating Depression Scale (SDS) with total score of 100 (50–59: mild; 60–69: moderate; >69: severe)
Sleep Quality: Pittsburgh Sleep Quality Index (PSQI) was used to assess sleep disturbances (score range: 0–21; higher scores indicate worse sleep quality)
Urinary Function: International Consultation on Incontinence Questionnaire-Short Form (ICIQ-SF) was used to evaluate urinary incontinence severity (score range: 0–21; higher scores indicate more severe symptoms)

Patient Satisfaction: Institutional questionnaire (total score: 100) categorizing satisfaction as:

Very satisfied: ≥90 points
Satisfied: 70–89 points
Dissatisfied: <70 points Overall satisfaction rate = (Very satisfied + Satisfied cases)/Total cases × 100%

### Quality assessment and evidence certainty

The quality of included studies and overall evidence was assessed using the Newcastle-Ottawa Scale (NOS) for observational studies. Additionally, we applied the Grading of Recommendations Assessment, Development and Evaluation (GRADE) approach to assess the certainty of evidence for each outcome. The GRADE assessment considered study limitations, inconsistency, indirectness, imprecision, and publication bias.

### Statistical analysis

Statistical analyses were performed using SPSS version 26.0 (IBM Corp., Armonk, NY, USA). Normally distributed continuous variables were expressed as mean ± standard deviation and compared using independent *t*-tests. Non-normally distributed data were presented as median (interquartile range) and analyzed using Wilcoxon rank-sum tests. Categorical variables were expressed as frequencies or percentages and compared using chi-square or Fisher's exact tests. Multivariate logistic regression was employed to identify independent risk factors. ROC curve analysis was performed using MedCalc version 20.0. Statistical significance was set at *P* < 0.05.

For missing data, we first conducted a comprehensive assessment across all variables. For continuous variables, when less than 5% of the data was missing, we imputed the missing values using the mean (for normally distributed data) or the median (for non—normally distributed data). For categorical variables with missing data, we used the mode to fill in the missing values. In cases where more than 5% of the data was missing for a particular variable, we excluded that variable from the analysis to prevent potential biases. Additionally, we carried out sensitivity analyses by repeating the main analyses with and without the imputed data. The results of these sensitivity analyses were consistent with our main findings, suggesting that the handling of missing data did not have a significant impact on our overall conclusions.

In our study, given the nature of our analysis, we did not perform multiple comparisons in the traditional sense where a large number of pairwise comparisons are made. Our primary focus was on identifying independent risk factors through univariate and multivariate analysis, and comparing outcome measures between two groups (the observation and control groups). For these group comparisons, we used appropriate statistical tests (independent t—tests for continuous variables and chi—square/Fisher's exact tests for categorical variables) with a significance level of *P* < 0.05. Since we did not conduct a series of multiple comparisons that would inflate the type I error rate, no specific adjustments such as Bonferroni correction were applied. However, we acknowledge that in future studies with a larger number of comparisons, appropriate adjustments for multiple testing would be necessary to ensure the validity of the results.

## Results

### Patient characteristics and screening

Of the 152 patients screened, 140 met the inclusion criteria and were included in the analysis (Figure 1). The excluded patients comprised: 5 with incomplete clinical documentation, 4 with chronic pain history, 2 with severe comorbidities, and 1 who declined participation.

### Univariate analysis of factors affecting analgesic efficacy after TURP

Univariate analysis revealed that several factors were significantly different between the poor and good analgesic response groups. The poor analgesic response group had longer operation times, and higher proportions of American Society of Anesthesiologists (ASA) class II patients, smokers, and those with hypertension or diabetes. The results showed that the poor analgesic response group had significantly longer operation times (81.25 ± 10.46 vs. 52.76 ± 9.87 min, *P* < 0.001) and higher proportions of ASA class II patients (55.00% vs. 40.00%, *P* = 0.028), smokers (85.00% vs. 28.33%, *P* < 0.001), and individuals with hypertension (66.25% vs. 40.00%, *P* = 0.034) or diabetes (40.00% vs. 36.67%, *P* = 0.038) (all *P* < 0.05). These variables—operation time, ASA classification, smoking history, hypertension, and diabetes—showed statistical significance in the univariate analysis and were thus selected for further multivariate logistic regression analysis as they were potentially associated with the perioperative analgesic efficacy.

### Multivariate analysis and model development

Before conducting multivariate analysis, we assessed multicollinearity among variables. The VIF values were: operation time (1.23), ASA classification (2.87), smoking history (1.15), hypertension (2.45), and diabetes (2.32). Since all VIF values were <5, no significant multicollinearity was detected, and all variables were retained in the model.

Multivariate logistic regression analysis identified prolonged operation time and smoking history as independent risk factors for inadequate perioperative analgesia in TURP patients. ASA classification, hypertension, and diabetes did not remain significant in the multivariate model, suggesting their effects may be mediated through other factors or confounded by the independent risk factors.

Regarding the comparison of outcome measures between the observation and control groups, the observation group had significantly lower post—intervention scores for pain (VAS), anxiety, depression, sleep disturbance, and urinary incontinence compared to the control group. This indicated that the mind map—guided nursing intervention was effective in reducing pain, improving psychological status, enhancing sleep quality, and alleviating urinary incontinence. Additionally, the nursing satisfaction rate was significantly higher in the observation group, showing greater patient satisfaction with the mind map—guided nursing care (Table 1).

**Table 1:** Univariate analysis of adverse analgesic effect after TURP operation.

Index	Good analgesia group	Poor analgesia group	*t*/*χ*^2^ value	*P* value
Cases	*n* = 60	*n* = 80		
Age (year)	62.34 ± 11.78	62.87 ± 12.04	*t* = 0.265	0.741
Gender			*χ*^2^=0.873	0.136
Male	22 (36.67%)	28 (35.00%)		
Female	38 (63.33%)	52 (65.00%)		
BMI (kg/m^2^)	21.37 ± 3.26	21.45 ± 3.18	*t* = 0.314	0.687
Time of operation (min)	52.76 ± 9.87	81.25 ± 10.46	*t* = 21.782	<0.001
ASA classification of anesthesia			*χ*^2^ = 5.487	0.028
Ⅰ	36 (60.00%)	36 (45.00%)		
Ⅱ	24 (40.00%)	44 (55.00%)		
Analgesia			*χ*^2^ = 0.365	0.638
Paravertebral block	25 (41.67%)	35 (43.75%)		
Epidural analgesia	35 (58.33%)	45 (56.25%)		
History of smoking			*χ*^2^ = 10.563	<0.001
Yes	17 (28.33%)	68 (85.00%)		
No	43 (71.67%)	12 (15.00%)		
History of drinking			*χ*^2^ = 0.832	0.162
Yes	27 (45.00%)	32 (40.00%)		
No	33 (55.00%)	48 (60.00%)		
Hypertension			*χ*^2^ = 5.143	0.034
Yes	24 (40.00%)	53 (66.25%)		
No	36 (60.00%)	27 (33.75%)		
Diabetes			*χ*^2^ = 5.012	0.038
Yes	22 (36.67%)	32 (40.00%)		
No	38(63.33%)	48(60.00%)		

### Multivariate logistic regression analysis of analgesic efficacy after TURP

Using poor analgesic effect at 72 h post—surgery as the dependent variable, we performed a multivariate logistic regression analysis on the factors that were significant in the univariate analysis. The factors included in the final regression model were prolonged operation time and smoking history. The reason for their inclusion is that they were identified as independent risk factors for inadequate perioperative analgesia in TURP patients through the regression analysis. The results identified prolonged operation time (OR = 1.528, 95% CI: 1.218–1.982, *P* = 0.013) and smoking history (OR = 1.278, 95% CI: 1.042–1.826, *P* = 0.038) as independent risk factors for inadequate perioperative analgesia in TURP patients (Table 2).

**Table 2:** Multivariate logistic regression analysis of adverse analgesic effect after TURP.

Index	*β* value	SE value	*P* value	OR value	95% CI
Time of operation	0.318	0.157	0.013	1.528	1.218–1.982
ASA classification of anesthesia	1.245	0.117	0.065	3.257	1.014–8.732
History of smoking	0.521	0.267	0.038	1.278	1.042–1.826
Hypertension	0.731	0.553	0.073	4.128	0.145–10.762
Diabetes	1.283	0.586	0.081	2.469	1.124–8.965

### Predictive model performance

**Figure 3:**
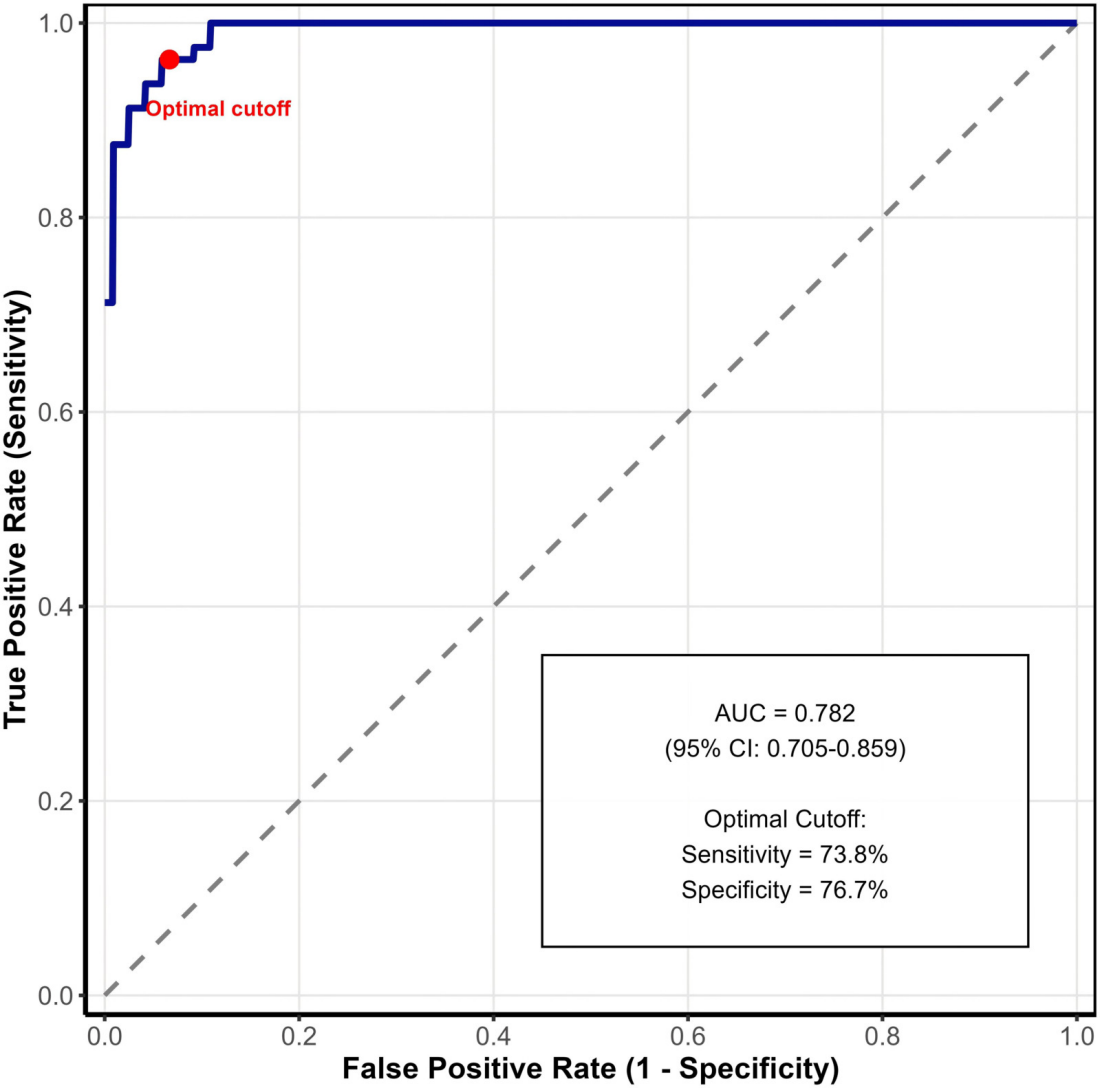
ROC Curve for Predictive Model of Poor Analgesic Response.

Based on the two independent risk factors (operation time and smoking history), we developed a predictive model for poor analgesic response. The ROC curve analysis showed an AUC of 0.782 (95% CI: 0.705–0.859, *P* < 0.001), indicating good discriminative ability. The optimal cut-off value yielded a sensitivity of 73.8% and specificity of 76.7% (Figure 3).

### Comparison of VAS scores between groups

Pre-intervention VAS scores showed no significant difference between groups (*P* > 0.05). Post-intervention, the observation group demonstrated significantly lower VAS scores compared to the control group (5.26 ± 0.41 vs. 7.02 ± 0.58, *P* = 0.005) (Table 3).

**Table 3:** Comparison of VAS scores between groups.

Groups	*n*	Pre-intervention	Post-intervention
Control	40	8.28 ± 0.73	7.02 ± 0.58
Observation	40	8.21 ± 0.69	5.26 ± 0.41^a^
*t* value		0.763	6.784
*P* value		0.342	0.005

a *P* < 0.05 compared with control group. Data presented as mean ± SD.

### Comparison of anxiety and depression scores

Initial anxiety and depression scores were comparable between groups (*P* > 0.05). Following intervention, the observation group showed significantly reduced anxiety (41.58 ± 3.42 vs. 52.41 ± 4.25, *P* = 0.007) and depression scores (41.26 ± 3.28 vs. 52.41 ± 4.19, *P* = 0.002) compared to the control group (Table 4).

**Table 4:** Comparison of anxiety and depression scores between groups.

Groups	*n*	Anxiety scores		Depression scores	
		Pre	Post	Pre	Post
Control	40	63.15 ± 5.47	52.41 ± 4.25	63.45 ± 4.57	52.41 ± 4.19
Observation	40	62.94 ± 5.63	41.58 ± 3.42*	63.88 ± 4.72	41.26 ± 3.28^a^
*t* value		0.725	5.821	0.718	7.156
*p* value		0.283	0.007	0.289	0.002

a *P* < 0.05 compared with control group. Data presented as mean ± SD.

### Comparison of nursing satisfaction

The observation group demonstrated significantly higher overall nursing satisfaction rates compared to the control group (95.00% vs. 75.00%, *P* = 0.003) (Table 5).

**Table 5:** Comparison of nursing satisfaction between groups.

Groups	*n*	Very satisfied	Satisfied	Unsatisfied	Overall satisfaction
Control	40	18 (45.00)	12 (30.00)	10 (25.00)	30 (75.00)
Observation	40	28 (70.00)	10 (25.00)	2 (5.00)	38 (95.00)^a^
*χ*2 value					6.124
*P* value					0.003

a *P* < 0.05 compared with control group. Data presented as *n* (%).

### Comparison of sleep quality

Pre-intervention PSQI scores were similar between groups (*P* > 0.05). Post-intervention, the observation group showed significantly improved sleep quality scores compared to the control group (5.42 ± 0.28 vs. 8.02 ± 0.24, *P* = 0.009) (Table 6).

**Table 6:** Comparison of sleep quality scores between groups.

Groups	*n*	Pre-intervention	Post-intervention
Control	40	13.45 ± 0.42	8.02 ± 0.24
Observation	40	13.18 ± 0.39	5.42 ± 0.28^a^
*t* value		0.573	5.764
*P* value		0.436	0.009

a *P* < 0.05 compared with control group. Data presented as mean ± SD.

### Comparison of urinary function

Pre-intervention ICIQ-SF scores showed no significant difference between groups (*P* > 0.05). Post-intervention, the observation group demonstrated significantly lower urinary incontinence scores compared to the control group (9.02 ± 1.14 vs. 13.21 ± 1.43, *P* = 0.001) (Table 7).

**Table 7:** Comparison of urinary incontinence scores between groups.

Groups	*n*	Pre-intervention	Post-intervention
Control	40	18.34 ± 2.13	13.21 ± 1.43
Observation	40	18.19 ± 2.05	9.02 ± 1.14^a^
*t* value		0.781	7.365
*P* value		0.328	0.001

a *P* < 0.05 compared with control group. Data presented as mean ± SD.

### Quality of evidence

According to GRADE assessment, the certainty of evidence was rated as moderate for pain outcomes and psychological outcomes, mainly due to the retrospective design and relatively small sample size. The evidence for sleep quality and urinary function outcomes was rated as low to moderate due to similar limitations plus the subjective nature of some assessment tools.

## Discussion

Our study identified prolonged operation time and smoking history as independent risk factors for inadequate perioperative analgesia in TURP patients. This finding is consistent with previous research. Peng et al. **(**6) found that the operation time to be associated with increased pain in TURP patients, while multiple studies have explored the relationship between smoking and analgesic efficacy, including Akanbi et al. (17) who demonstrated reduced analgesic responses in chronic smokers. Regarding the mind map—guided nursing intervention, we compare our results with those of Jaguga et al. (18) and Younas and Quennell (19), which have also evaluated the impact of such interventions on pain and psychological outcomes. For instance, studies have shown that longer surgical procedures increase tissue trauma, leading to greater release of inflammatory mediators and subsequent pain amplification (16). Our results further confirm this relationship, with a significant odds ratio for operation time (OR = 1.528, 95% CI: 1.218–1.982). Regarding smoking history, our finding that it is an independent risk factor aligns with the understanding that nicotine can modulate pain perception through its effects on narcotic drug dependence (20). Chronic smoking may lead to desensitization of analgesic responses, as reflected in our results with an OR of 1.278 (95% CI: 1.042–1.826).

### Clinical implications and mechanistic insights

The clinical implications of our findings are substantial. The identification of prolonged operation time and smoking history as independent risk factors allows for preoperative risk stratification and targeted interventions. For patients with these risk factors, clinicians might consider: (1) enhanced preoperative optimization, including smoking cessation programs initiated well before surgery; (2) modified anesthetic protocols with multimodal analgesia approaches; (3) more intensive postoperative monitoring and pain management strategies; (4) early implementation of mind map-guided nursing interventions for high-risk patients.

The mechanism by which prolonged operative time affects perioperative analgesic efficacy is complex. Longer surgical procedures mean more extensive tissue manipulation by surgical instruments. This extended interaction directly damages cells and triggers a series of biological responses. The immune system is activated, leading to the release of cytokines such as interleukin-1 (IL-1), interleukin-6 (IL-6), and tumor necrosis factor-α (TNF-α). These cytokines play a key role in the inflammatory process. IL-1 and TNF-α can directly sensitize pain receptors, like the transient receptor potential vanilloid 1 (TRPV1) receptors, making them more responsive to pain stimuli. IL-6, on the other hand, can modulate the immune response and also contribute to the amplification of pain signals. Moreover, the longer the operation, the more analgesic medication is often required. However, increased use of analgesics can lead to more side effects, such as respiratory depression, nausea, and vomiting. These side effects can further complicate pain management and overall patient recovery.

### The mechanistic basis of mind map-guided nursing

The effectiveness of mind map-guided nursing interventions can be explained through several theoretical frameworks. From a cognitive load theory perspective, mind maps reduce the cognitive burden of processing complex medical information by presenting it in a visually organized manner (21). This is particularly important for postoperative patients who may experience cognitive impairment due to anesthesia and pain medications. The dual coding theory suggests that information presented both visually and verbally (as in mind maps) is better retained and recalled (22). Furthermore, the patient engagement theory posits that interactive visual tools enhance patient participation in their care, leading to better adherence to treatment protocols and improved outcomes (23).

In terms of the mind map—guided nursing interventions, our study demonstrated significant improvements in pain levels, psychological status, sleep quality, and urinary incontinence, as well as increased nursing satisfaction. Similar to our findings, Wang et al. (24) reported that patients in the mind map—guided nursing group had better pain management knowledge and lower pain scores. He et al. (25) also found that such interventions enhanced communication between nurses and patients, leading to improved pain control. Our study extends these findings by specifically evaluating the impact on TURP patients, a population with unique postoperative challenges.

The positive impact of mind map—guided nursing on psychological and pain outcomes can be attributed to several factors. Firstly, the visual nature of mind maps simplifies complex medical information. By presenting information in a hierarchical and visual format, patients can more easily understand their condition, treatment procedures, and pain management strategies. This enhanced understanding reduces anxiety and fear related to the unknown, thus improving their psychological state. For example, patients can clearly visualize the connection between their surgical procedures, potential pain sources, and the corresponding pain relief measures. Secondly, the individualized approach in mind map—guided nursing plays a crucial role. The care plan is customized based on each patient's specific needs, such as their medical history, pain tolerance, and psychological status. This personalized care can better address patients' unique pain triggers and psychological concerns. For patients with a history of anxiety, the mind map can include specific relaxation techniques and coping strategies. Thirdly, the implementation of mind map—guided nursing promotes enhanced communication between nurses and patients. Nurses can use the mind map as a visual aid to explain treatment plans and answer patients' questions more effectively. This increases patients' trust in the healthcare team and their compliance with treatment, ultimately leading to better pain management and overall psychological well—being.

### Limitations and future directions

While our study provides valuable insights, several limitations must be acknowledged. The retrospective design inherently limits causal inference, and the single-center nature may affect generalizability. The relatively small sample size (*n* = 140) may have limited our ability to detect smaller but clinically significant effects. Additionally, we did not assess long-term outcomes beyond the immediate postoperative period, which would be valuable for understanding the sustained benefits of mind map-guided interventions.

Future research should address these limitations through: (1) prospective, multicenter randomized controlled trials with larger sample sizes; (2) long-term follow-up to assess sustained benefits and potential late complications; (3) Investigation of optimal timing and intensity of mind map-guided interventions; (4) development of standardized mind map protocols for different surgical populations; (5) health economic analysis to evaluate cost-effectiveness of implementation; (6) exploration of digital mind mapping tools and their integration with electronic health records.

Minimally invasive surgical techniques have revolutionized modern medical practice. Compared to traditional open procedures, minimally invasive approaches offer distinct advantages, including reduced operative time, smaller incisions, and diminished postoperative pain (8, 9). While optimal analgesic management can minimize analgesia-related complications and expedite functional recovery, achieving complete pain elimination remains challenging (10, 11).

Inadequate postoperative pain control can trigger a cascade of adverse outcomes. These include sympathetic nervous system activation, increased systemic oxygen consumption, postoperative hypercoagulation, and immunosuppression. Additionally, patients may experience complications such as atelectasis, pneumonia, and hypercapnia. These complications can lead to prolonged hospitalization, diminished quality of life, and increased healthcare costs (12, 13). Surgical trauma represents the primary etiological factor in postoperative pain. Research has demonstrated that surgical interventions can initiate peripheral and central nerve pain sensitization through incisional trauma and inflammatory mediator activation, resulting in acute postoperative pain (13, 14). Without adequate pain management, peripheral nerve endings sensitization and central nerve sensitization may develop, potentially leading to chronic pain syndrome in some patients (15, 16).

Our multivariate logistic regression analysis identified prolonged operation time and smoking history as independent risk factors for inadequate perioperative analgesia following TURP. The correlation between extended operative time and increased pain may be attributed to several factors. Surgical procedures inherently cause tissue trauma, triggering immune mechanism activation and subsequent inflammatory mediator release, which can amplify pain sensation (16). Longer operative times typically correspond to greater surgical trauma and enhanced inflammatory mediator release. Conversely, shorter operative times generally result in reduced stress responses and postoperative pain intensity. Furthermore, minimizing operative time can reduce the required analgesic medication, thereby decreasing the risk of adverse effects such as respiratory depression, nausea, and vomiting (26, 27). Surgeons can potentially improve outcomes by enhancing surgical technique efficiency and selecting straightforward surgical approaches to minimize operative time and reduce physiological stress responses (28, 29).

Patients with chronic conditions such as diabetes and hypertension present unique challenges in pain management. Diabetes can compromise immune system function and metabolic regulation, particularly in cases of prolonged illness or poor glycemic control. Hypertensive patients often experience vascular endothelial dysfunction, resulting in decreased production of protective endothelial factors and reduced compensatory stress response capabilities. These pathophysiological changes may contribute to diminished surgical tolerance and more severe postoperative pain under standard analgesic protocols (20, 29, 30).

The impact of smoking on analgesic efficacy appears to be mediated through nicotine's effects on narcotic drug dependence. Nicotine acts on specific receptors, including nicotinic acetylcholine receptors, and can modulate pain perception through spinal cord mechanisms or cholinergic microglial pathways (20). Chronic exposure may lead to desensitization of analgesic responses, resulting in reduced efficacy of standard postoperative analgesic protocols and increased analgesic requirements (31).

The implementation of mind map-guided nursing interventions demonstrated significant improvements in patient outcomes, as evidenced by reduced VAS scores, anxiety and depression scores, sleep quality scores, and urinary incontinence scores in the observation group. Mind map-guided nursing represents a patient-centered approach to healthcare delivery. This methodology employs visual mapping techniques to present complex medical information in an accessible, network-diagram format, facilitating patient comprehension and retention of health-related knowledge (32–35). The enhanced understanding achieved through mind map guidance appears to stabilize patients' psychological state and improve treatment compliance, fostering a more collaborative nurse-patient relationship that ultimately supports better rehabilitation outcomes (32, 36–38).

This study has notable limitations. Its retrospective design may introduce selection bias as it uses pre—collected data, and patients were not randomly assigned. The sample size of 140 patients is relatively small, limiting statistical power and potentially missing small but significant effects. There are also several confounding factors. Surgical techniques vary among surgeons, and postoperative care protocols may differ across units, both of which could impact outcomes. Additionally, unmeasured patient—specific factors like genetic predisposition and lifestyle might have influenced the results. These limitations call for caution when interpreting our findings. Future research should consider prospective designs, larger samples, and better control of confounding factors to further explore these aspects.

## Conclusion

This study demonstrates that multiple factors influence postoperative analgesic efficacy in patients undergoing TURP. Our findings reveal significant correlations between postoperative analgesia and several clinical parameters, including operative time, ASA anesthesia grade, smoking history, and comorbid conditions. Through multivariate analysis, we identified prolonged operative time and smoking history as independent risk factors for suboptimal analgesic outcomes. The development of a predictive model with good discriminative ability (AUC = 0.782) provides a practical tool for preoperative risk assessment. Mind map-guided nursing interventions demonstrated significant efficacy in improving multiple patient outcomes, including pain control, psychological well-being, sleep quality, and urinary function. These findings support the integration of visual nursing tools in perioperative care protocols for TURP patients, particularly those identified as high-risk for inadequate pain control.

## Data Availability

The original contributions presented in the study are included in the article/Supplementary Material, further inquiries can be directed to the corresponding author.

## References

[1] Chai Y Zhou Z Cui Y Che X Zhang Y. Outcomes and complications of naftopidil versus tamsulosin for elderly men with lower urinary tract symptoms secondary to benign prostatic hyperplasia: a systematic review and meta-analysis. Andrologia. (2021) 53(9):e14166. 10.1111/and.14166 34189764

[2] Langan RC. Men’s health: benign prostatic hyperplasia. FP Essent. (2021) 503:18–22. 33856179

[3] Licari LC Bologna E Manfredi C Franco A Ditonno F De Nunzio C Incidence and management of BPH surgery-related urethral stricture: results from a large U.S. database. Prostate Cancer Prostatic Dis. (2024) 27(3):537–43. 10.1038/s41391-024-00841-z 38714780

[4] Zhang J Che J Sun X Ren W. Effect of intravenous injection of remazolam on stress response and analgesic effect in patients with transurethral resection of the prostate: a single-centre study. Arch Esp Urol. (2023) 76(10):780–6. 10.56434/j.arch.esp.urol.20237610.94 38186071

[5] Atim T Obiatuegwu KO. Monopolar transurethral resection of the prostate by a single surgeon in North-Central Nigeria: surgical results and postoperative complications. West Afr J Med. (2024) 41(4):421–8. 39003514

[6] Peng YN Jin L Peng EJ Zhang L. Perioperative care based on roy adaptation model in elderly patients with benign prostatic hyperplasia: impact on psychological well-being, pain, and quality of life. BMC Urol. (2023) 23(1):172. 10.1186/s12894-023-01343-1 37891515 PMC10612228

[7] Ma Y Wang Y Liu H Zhang Q Hu P. Effect of ropivacaine combined with nalbuphine on pain during anaesthesia recovery in patients with prostatic hyperplasia undergoing transurethral resection of prostate. Arch Esp Urol. (2024) 77(7):746–52. 10.56434/j.arch.esp.urol.20247707.104 39238298

[8] Chung JM Ha HK Kim DH Joo J Kim S Sohn DW Evaluation of the efficacy of solifenacin for preventing catheter-related bladder discomfort after transurethral resection of bladder tumors in patients with non-muscle invasive bladder cancer: a prospective, randomized, multicenter study. Clin Genitourin Cancer. (2017) 15(1):157–62. 10.1016/j.clgc.2016.05.006 27346074

[9] Zhou J Peng ZF Song P Yang LC Liu ZH Shi SK Enhanced recovery after surgery in transurethral surgery for benign prostatic hyperplasia. Asian J Androl. (2023) 25(3):356–60. 10.4103/aja202267 36254889 PMC10226510

[10] Li C Ji F Fan F Xu J Xu H. Transperineal Botulinum toxin injection for chronic pelvic pain syndrome after transurethral resection of the prostate. Urol J. (2022) 19(4):333–8. 10.22037/uj.v19i.7128 35762081

[11] Chattopadhyay I Banerjee SS Jha AK Basu S. Effects of intrathecal dexmedetomidine as an additive to low-dose bupivacaine in patients undergoing transurethral resection of prostate. Indian J Anaesth. (2017) 61(12):1002–8. 10.4103/ija.IJA_324_16 29307907 PMC5752769

[12] Charoenpol FN Khampitak N Aimnang C Pachirat K Sirithanaphol W Rompsaithong U Single-dose intravenous nefopam on postoperative catheter-related bladder discomfort in patients undergoing transurethral resection of prostate: a randomized, double-blind placebo-controlled trial. J Anesth. (2023) 37(1):72–8. 10.1007/s00540-022-03130-y 36319912

[13] Li L Li S Sun Y Zhang S Zhang X Qu H. Personalized preoperative education reduces perioperative anxiety in old men with benign prostatic hyperplasia: a retrospective cohort study. Gerontology. (2021) 67(2):177–83. 10.1159/000511913 33454707

[14] Lu P Wu C. Continuous psychological nursing based on grey clustering algorithm in patients after transurethral resection of prostate. Comput Math Methods Med. (2022) 2022:5400479. 10.1155/2022/5400479 35936363 PMC9352487

[15] Yan Y Yuehong W Kun L Hongbo Z Hongyu Z Yingming Y Implementation of mind mapping with problem-based learning in prosthodontics course for Chinese dental students. BMC Med Educ. (2023) 23(1):530. 10.1186/s12909-023-04479-8 37491283 PMC10369705

[16] Rühle A Blarer J Oehme F Marini L Mattei A Stucki P Safety and effectiveness of bipolar transurethral resection of the prostate in patients under ongoing oral anticoagulation with coumarins or antiplatelet drug therapy compared to patients without anticoagulation/antiplatelet therapy. J Endourol. (2019) 33(6):455–62. 10.1089/end.2018.0879 30834782

[17] Akanbi MO Carroll AJ Achenbach C O’Dwyer LC Jordan N Hitsman B The efficacy of smoking cessation interventions in low- and middle-income countries: a systematic review and meta-analysis. Addiction. (2019) 114(4):620–35. 10.1111/add.14518 30506845 PMC6411424

[18] Jaguga F Kiburi SK Temet E Aalsma MC Ott MA Maina RW A scoping review of substance use brief interventions in Africa. PLOS Glob Public Health. (2024) 4(10):e0003340. 10.1371/journal.pgph.0003340 39446874 PMC11501030

[19] Younas A Quennell S. Usefulness of nursing theory-guided practice: an integrative review. Scand J Caring Sci. (2019) 33(3):540–55. 10.1111/scs.12670 30866078

[20] Wang J Fu G Liu J Yu Y Wang N. Effect of preoperative gabapentin after transurethral prostate resection under general anesthesia. A randomized double-blind, placebo-controlled trial. Saudi Med J. (2020) 41(6):640–4. 10.15537/smj.2020.6.25132 32518932 PMC7502946

[21] Sweller J van Merrienboer JJG Paas FGWC. Cognitive architecture and instructional design: 20 years later. Educ Psychol Rev. (2019) 31(2):261–92. 10.1007/s10648-019-09465-5

[22] Paivio A. Mental Representations: A Dual Coding Approach. Oxford: Oxford University Press (2013).

[23] Graffigna G Barello S Bonanomi A Lozza E. Measuring patient engagement: development and psychometric properties of the patient health engagement (PHE) scale. Front Psychol. (2015) 6:274. 10.3389/fpsyg.2015.00274 25870566 PMC4376060

[24] Wang G Xia Y Halili X Tang S Chen Q. Academic-practice partnerships in evidence-based nursing education: protocol of a theory-guided scoping review. Nurse Educ Pract. (2023) 69:103644. 10.1016/j.nepr.2023.103644 37058995

[25] He Y Chen J Chen Y Qian H. Effect of operating room nursing management on nosocomial infection in orthopedic surgery: a meta-analysis. J Healthc Eng. (2022) 2022:4193932. 10.1155/2022/4193932 35256898 PMC8898123

[26] Köse O Sağlam HS Altun ME Sonbahar T Kumsar Ş Adsan Ö. Prilocaine irrigation for pain relief after transurethral resection of the prostate. J Endourol. (2013) 27(7):892–5. 10.1089/end.2013.0001 23565930

[27] Kwon SY Joo JD Cheon GY Oh HS In JH. Effects of dexmedetomidine infusion on the recovery profiles of patients undergoing transurethral resection. J Korean Med Sci. (2016) 31(1):125–30. 10.3346/jkms.2016.31.1.125 26770048 PMC4712570

[28] Wang H Deng W Zhu X Fei C. Perioperative analgesia with ultrasound-guided quadratus lumborum block for transurethral resection of prostate. Medicine (Baltimore). (2021) 100(51):e28384. 10.1097/MD.0000000000028384 34941168 PMC8702260

[29] Doger C Yüksel BE Canoler O Ornek D Emre C Kahveci K. Effects of intrathecal bupivacaine and bupivacaine plus sufentanil in elderly patients undergoing transurethral resection. Niger J Clin Pract. (2014) 17(2):149–53. 10.4103/1119-3077.127423 24553022

[30] Gup DI Shapiro E Baumann M Lepor H. Contractile properties of human prostate adenomas and the development of infravesical obstruction. Prostate. (1989) 15(2):105–14. 10.1002/pros.2990150204 2477832

[31] Donahue RP Stamm AW Daily AM Kozlowski PM Porter CR Govier FE Opioid-limiting pain control after transurethral resection of the prostate: a randomized controlled trial. Urology. (2022) 166:202–8. 10.1016/j.urology.2022.03.010 35314185

[32] Hattori Y Kambe T Mine Y Hagimoto H Kokubun H Abe Y Transvesical bladder diverticulectomy via bladder neck opening during robot-assisted radical prostatectomy. Asian J Endosc Surg. (2024) 17(3):e13318. 10.1111/ases.13318 38716571

[33] Li H Li Y He R. Sparing effects of sufentanil on epidural ropivacaine in elderly patients undergoing transurethral resection of prostate surgery. Yonsei Med J. (2015) 56(3):832–7. 10.3349/ymj.2015.56.3.832 25837193 PMC4397457

[34] Lang Ben Nun E Sela HY Joseph J Rudelson G Grisaru-Granovsky S Rottenstreich M. Prolonged operative time of cesarean is a risk marker for subsequent cesarean maternal complications. Arch Gynecol Obstet. (2022) 307(3):739–46. 10.1007/s00404-022-06575-4 35488051

[35] Lyu X Zheng D Zhang H Zhang T Han N Zhang M Hyperthermia improves immune function and radiotherapy efficacy in patients with post-operative recurrent gastric cancer. Hepato-gastroenterology. (2014) 61 (136):2428–33. 25699397

[36] Goldstein SA Yu S Lowery R Halligan NL Dahmer MK Rocchini A. Analysis of inflammatory cytokines in the chest tube drainage of post-operative superior cavopulmonary connection patients. Cardiol Young. (2022) 33(6):925–32. 10.1017/S1047951122001913 35766168

[37] Dias Quintão JL Reis Gonzaga AC Galdino G Lima Romero TR Silva J Lemos V TNF-α, CXCL-1 and IL-1 β as activators of the opioid system involved in peripheral analgesic control in mice. Eur J Pharmacol. (2021) 896:173900. 10.1016/j.ejphar.2021.173900 33545158

[38] Hunter CA Jones SA. IL-6 as a keystone cytokine in health and disease. Nat Immunol. (2015) 16(5):448–57. 10.1038/ni.3153 25898198

